# Crimean-Congo Hemorrhagic Fever Beyond Ribavirin: A Systematic Review

**DOI:** 10.7759/cureus.17842

**Published:** 2021-09-09

**Authors:** Stephanie P Fabara, Juan Fernando Ortiz, Derrick Wayne Smith, Jashank Parwani, Sashwath Srikanth, Teresa Varghese, Maria Paez, Prarthana Desai, Raghavendra Tirupathi

**Affiliations:** 1 Internal Medicine, Universidad Catolica de Santiago de Guayaquil, Guayaquil, ECU; 2 Neurology, Universidad San Francisco de Quito, Quito, ECU; 3 Neurology, Larkin Community Hospital, Miami, USA; 4 Health Sciences, American University of the Caribbean, Pembroke Pines, USA; 5 Neurology, Lokmanya Tilak Municipal Medical College, Mumbai, IND; 6 Internal Medicine, SRM Medical College Hospital and Research Center, Chennai, IND; 7 Medicine, Kasturba Medical College, Manipal University, Manipal, IND; 8 General Medicine, Pontificia Universidad Catolica del Ecuador, Quito, ECU; 9 Internal Medicine, Gujarat Medical Education & Research Society (GMERS) Medical College, Vadodara, IND; 10 Internal Medicine, Keystone Health, Chambersburg, USA

**Keywords:** crimean congo hemorrhagic fever, therapeutic plasmapheresis, dexamethasone, treatment choices, intravenous immunoglobulin (ivig), hyperimmunoglobulin

## Abstract

Crimean-Congo hemorrhagic fever (CCHF) is a tick-borne virus endemic to a vast geographical area spanning from Africa to the shores of the Mediterranean Sea and north to the Balkans. The infection carries a high case fatality rate, which prompts the development of new treatment and prophylactic measures. This review explores the different treatment and prophylactic measures found in recent literature. For this purpose, we used Medical Subject Headings (MeSH) as well as PubMed advanced search. The inclusion criteria included full-text studies conducted on humans and in the English language. We found that plasma exchange was associated with a decrease in mortality rates. Similarly, the use of immunoglobulins proved effective in decreasing the severity and mortality risk. Ribavirin use was determined as a post-exposure prophylaxis drug with no statistically significant difference in oral or intravenous routes of administration. More studies should be conducted on CCHF as the number of outbreaks and endemic areas seem to be on the rise. For the time being, supportive therapy along with adjuvant antivirals appear to be the main course of management of CCHF. However, the need for definitive therapeutic agents and guidelines is warranted.

## Introduction and background

Crimean-Congo hemorrhagic fever (CCHF), caused by a tick-borne virus of genera *Nairovirus* and family *Bunyaviridae*, is endemic in 47 countries in Eastern and Southern Europe, Northwestern China, Central Asia, Africa, the Middle East, and the Indian subcontinent. The disease was first recognized in Crimea in 1944 and later in Congo in 1969 [1]. CCHF is the most widespread disease of all tick-borne viral diseases [2]. The virus is transmitted by the Hyalomma tick, which serves as both a reservoir and a vector. Human-to-human transmission occurs via direct contact or through bodily fluids during the incubation period of day 1 to 13. Health care workers (HCWs) and people working in close contact with animals are at risk of contracting the disease, with fatality rates ranging from 9% to 50% [3].

Clinical progression of the disease occurs via the following four phases: incubation, pre-hemorrhagic, hemorrhagic, and convalescence. The pre-hemorrhagic phase is acute and manifests as fever, headache, chills, nausea, vomiting, hyperemia, enanthemas, and rheumatic and lumbar pain. Diagnosis during this period is important for efficient management of the disease. The short and rapidly worsening hemorrhagic phase manifests as petechiae, ecchymosis, hematomas, or massive hemorrhages. The convalescence phase occurring after 15-20 days of onset of illness is characterized by general weakness, fatigability, poor appetite, nausea, poor vision and hearing, memory loss, and headache [4]. Diagnosis should be done by reverse transcription polymerase chain reaction (RT-PCR) during the period of infectivity. Serological diagnosis is done by enzyme-linked immunosorbent assay (ELISA) to detect IgM and IgG and has an excellent specificity [2,5]. Endothelial cells (ECs) and immune cells are likely targets in CCHF. One theory states that the virus stimulates ECs directly to release proinflammatory cytokines. In severe cases, this leads to increased vascular permeability, vasodilatation, and, subsequently, hypotension, multiple organ failure, shock, and death. CCHF may also block the immune response in several ways, such as only partial activation of dendritic cells and macrophages, decreased antibody response, apoptosis of lymphocytes, and hemophagocytosis. These can aid uncontrolled viral replication and systemic spread [6].

The treatment is primarily supportive. There are no treatment guidelines for CCHF based on the severity of illness. However, ribavirin is mainly used in practice during outbreaks because it has a higher degree of evidence. At the moment, there are two systematic reviews regarding the efficacy of ribavirin of CCHF [7,8]. In the first systematic review, ribavirin efficacy in clinical trials was inconclusive, although post-exposure prophylaxis (PEP) with ribavirin has shown some promising results in reducing the spread of virus, disease severity, and mortality [7]. Early administration of ribavirin proved to be beneficial in curbing the number of deaths among HCWs [8]. From a pooled analysis of a random clinical trial and an observational study, no significant difference was noticed in the mean length of hospital stay between patients on ribavirin and patients not on ribavirin [7].

In a systematic review by Ergönül et al., PEP among HCWs with ribavirin was effective. Overall, 7% of patients who received PEP contracted the infection in contrast to 89% who did not. The odds of infection reduced with ribavirin use (OR: 0.01; 95% CI: 0-0.03), and ribavirin initiation <48 hours after symptom onset reduced the odds of death (OR: 0.03; 95% CI: 0-0.58) [7].

Another systematic review by Soares-Weiser et al., involved 21 studies that showed that ribavirin treatment was not superior to no ribavirin treatment on clinical trials (RR: 1.13; 95% CI: 0.29-4.32). However, in observational studies, ribavirin was superior to no ribavirin treatment (RR: 0.56; 95% CI: 0.35-0.90). While ribavirin has proved to have some efficacy in two systematic reviews, the quality of those studies was poor according to the authors [7,9]. There are more drugs for the treatment of CCHF in clinical trials, observational studies, and case reports. Nevertheless, a detailed analysis of these treatments has not been conducted. We will perform a systematic review of the treatment of CCHF beyond ribavirin to consolidate the knowledge of CCHF regarding treatment and to establish a better strategy for treating the infection with other available therapeutic options.

## Review

Materials and Methods

A systematic review was conducted using the Preferred Reporting Items for Systematic Reviews and Meta-Analyses (PRISMA) and Meta-Analyses of Observational Studies in Epidemiology (MOOSE).

Eligibility Criteria and Study Selection

We solely included case reports, clinical trials, and observational studies that were conducted on humans, whereas animal studies were excluded. We also excluded any papers that did not meet our study objectives. After careful consideration, we included the papers with one of the following characteristics:

1) Patients: individuals with CCHF

2) Intervention: use of corticosteroids, immunoglobulins, plasmapheresis, or hyperimmunoglobulin

3) Comparator: placebo or control group

4) Outcomes: cure rate, mortality rate, case fatality rate, or duration of symptoms

Database and Search Strategy

We utilized the PubMed database for this systematic review. The search was conducted from June 28, 2021, to July 16, 2021. An advanced search was used with the following key terms: (Crimean-congo hemorrhagic fever[Title/Abstract]) AND (corticosteroids[Title/Abstract]) (Crimean-congo hemorrhagic fever[Title/Abstract]) AND (immunoglobulin[Title/Abstract]) (Crimean-congo hemorrhagic fever[Title/Abstract]) AND (plasmapheresis[Title/Abstract]) (Crimean-congo hemorrhagic fever[Title/Abstract]) AND (hyperimmunoglobulin[Title/Abstract]).

Data Extraction and Analysis

Data pertaining to the following information were extracted from each paper: title, author, year, country where the study was conducted, study type, methods, and outcomes.

Bias Assessment

**Figure 1:**
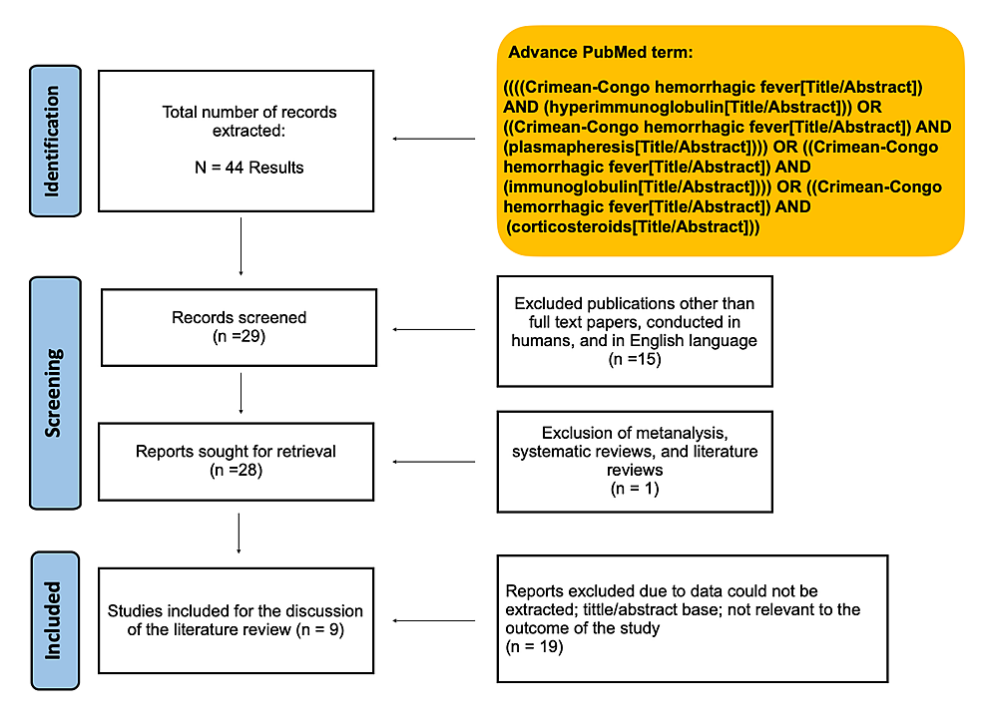
Flowchart of the extraction of data of the paper

We utilized the Risk of Bias in Non-Randomized Studies of Interventions (ROBINS-I) [10], the Cochrane collaboration risk-of-bias tool [11], and the Newcastle-Ottawa Scale (NOS) [12] to evaluate any bias in each of the studies. Figure 1 shows the PRISMA flow chart of the systematic review.

Results

Table 1 shows the study type, methods, outcomes, and the country of the studies that analyzed the use of plasma exchange for the treatment of CCHF [13-16].

**Table 1:** Results of the case reports and clinical trials of the systematic review CCHF, Crimean-Congo hemorrhagic fever; DIC, disseminated intravascular coagulation; ED, emergency department; ICU, intensive care unit; RT-PCR, reverse transcription polymerase chain reaction; SSI, severity score index

Author, year	Country	Study type	Number of patients	Methods	Outcome
Beştepe et al., 2021 [13]	Turkey	Clinical trial	119	Patients received either plasma exchange or standard care. They also were divided into mild, moderate, and severe according to SSI.	Lower mortality rate. Duration of hospitalization and platelet recovery was longer.
Ture and Kalin-Unuvar, 2020 [14]	Turkey	Case report	1	No particular methodology	A patient that was dealing with animal husbandry and had a tick bite history was admitted to the ED with complaints of high fever, nausea, and weakness. Laboratory findings showed bicytopenia, abnormal liver function tests, and elevated coagulation parameters. RT-PCR confirmed the diagnosis of CCHF. Three sessions of plasmapheresis were performed due to continued fever and worsening in laboratory values. Pulmonary embolism was detected in computerized tomography of the thorax carried out due to respiratory alkalosis on the sixth day. She was successfully treated with supportive and anticoagulation therapy.
Meço et al., 2013 [15]	Turkey	Case report	1	No particular methodology	A patient with CCHF developed leukopenia, thrombocytopenia, and liver failure. He received eight sessions of plasmapheresis on days 3, 4, 5, 7, 8, 9, 10, and 11. He was discharged from the ICU on day 16 with significant clinical improvement.
Kurnaz et al., 2011 [16]	Turkey	Case report	1	No particular methodology	A patient was hospitalized with high fever, mucosal bleeding, and decreased level of consciousness. His condition got complicated with thrombocytopenia and liver failure. At the ICU, he was given oral ribavirin without much improvement. He later developed DIC, and plasma exchange was given. The condition of this patient improved rapidly with the combined treatment.

Table 2 shows the study type, methods, outcomes, and the country of the studies that analyzed the use of corticosteroids for the treatment of CCHF [17,18].

**Table 2:** Results of the case report and observational study of the systematic review CCHF, Crimean-Congo hemorrhagic fever; SSI, severity score index

Author, year	Country	Study type	Number of patients	Methods	Outcome
Dokuzoguz et al., 2013 [17]	Turkey	Observational	281	Patients confirmed with CCHF received ribavirin and supportive treatment. If the condition didn’t improve, they administered dexamethasone. SSI was adjusted for confounding.	Decrease case fatality rate in high severity of the disease
Jabbari et al., 2006 [18]	Iran	Case report	6	No particular methodology	Mortality and cure with ribavirin and corticosteroids

Table 3 shows the study type, methods, outcomes, and the country of the studies that analyzed the use of intravenous immunoglobulin (IVIG) for the treatment of CCHF [19,20].

**Table 3:** Results of the clinical trials of the systematic review CCHF, Crimean-Congo hemorrhagic fever; FFP, fresh frozen plasma; HLH, hemophagocytic lymphohistiocytosis; IVIG, intravenous immunoglobulin

Author, year	Country	Study type	Number of patients	Methods	Outcome
Erduran et al., 2013 [19]	Turkey	Retrospective study	12	These patients were diagnosed with CCHF associated with HLH. There were 10 females and 2 males. They were successfully treated with methylprednisolone, FFP, and IVIG.	Improve recovery and reduce mortality
Salehi et al., 2013 [20]	Iran	Single blinded clinical trial	40	They compare the use of IVIG + ribavirin vs. ribavirin alone. The study included 12 cases and 28 controls. Patients who received IVIG had diminished severity of clinical signs and reduced disease duration; however, there was no statistical significance in mortality in both groups.	Improve recovery

Table 4 shows the study type, methods, outcomes, and the country of the studies that analyzed the use of hyperimmunoglobulin for the treatment of CCHF [21].

**Table 4:** Results of the clinical trial of the systematic review CCHF, Crimean-Congo hemorrhagic fever; KU, Kubar units; RT-PCR, reverse transcriptase polymerase chain reaction

Author, year	Country	Study type	Number of patients	Methodology	Results
Kubar et al., 2011 [21]	Turkey	Clinical trial	26	CCHF hyperimmunoglobulin product from 22 individuals who survived the infection was prepared. As a standard therapeutic approach, 400 KU of hyperimmunoglobulin was given to each patient as a single dose before viral load was detected. Also, they used one-step real-time RT-PCR to monitor the viral load of CCHF patients. According to the results, 15 patients with a viral load of 10^8^ copies/mL or more were defined as high risk.	The survival rate was 86% (13/15) among patients treated with hyperimmunoglobulin.

Bias Analysis

We used three different tools for assessing the bias of the systematic review: The Cochrane Collaboration’s tool for assessing risk of bias, the ROBINS-1 tool, and the NOS for assessing risk of bias in case reports.

Table 5 shows the bias analysis of the clinical trials.

**Table 5:** The Cochrane Collaboration’s tool for assessing risk of bias in clinical trials

	Random sequence generation	Allocation concealment	Blinding of participants and personnel	Blinding of outcome assessment	Incomplete outcome data	Selective reporting	Other bias
Beştepe et al., 2021 [13]	High risk	High risk	High risk	High risk	High risk	High risk	Unclear
Salehi et al., 2013 [20]	Low risk	Low risk	High risk	High risk	High risk	High risk	Unclear
Kubar et al., 2011 [21]	High risk	High risk	High risk	High risk	High risk	High risk	Unclear

Table 6 shows the bias analysis of the observational studies.

**Table 6:** ROBINS-I tool for assessing risk of bias in observational studies ROBINS, Risk of Bias in Non-Randomized Studies of Interventions

Study	Confounding	Selection of participants	Classification	Deviations	Missing data	Measurements	Selection of the reported results
Erduran et al., 2013 [19]	Intermediate risk	Intermediate risk	High risk	Low risk	Low risk	Intermediate risk	Low risk
Dokuzoguz et al., 2013 [17]	Low risk	High risk	Low risk	Low risk	Low risk	Low risk	Intermediate risk

Table 7 shows the bias analysis of the case reports.

**Table 7:** The Newcastle-Ottawa Scale for assessing risk of bias in case reports

Study	Ture and Kalin-Unuvar, 2020 [14]	Meço et al., 2013 [15]	Kurnaz et al., 2011 [16]	Jabbari et al., 2006 [18]
Selection				
Does the patient(s) represent(s) the whole experience of the investigator (center) or is the selection method unclear to the extent that other patients with similar presentation may not have been reported?	Yes	Yes	Yes	Yes
Ascertainment				
Was the exposure adequately ascertained?	Yes	Yes	Yes	Yes
Was the outcome adequately ascertained?	Yes	Yes	Yes	Yes
Causality				
Were other alternative causes that may explain the observation ruled out?	No	No	No	No
Was there a challenge/rechallenge phenomenon?	Yes	Yes	No	No
Was there a dose-response effect?	Unclear	Unclear	Yes	Unclear
Was follow-up long enough for outcomes to occur?	Yes	Yes	Yes	Yes
Reporting				
Is the case(s) described with sufficient details to allow other investigators to replicate the research or to allow practitioners make inferences related to their own practice?	Yes	Yes	Yes	Yes
Overall appraisal	High quality	High quality	High quality	High quality

Discussion

Plasma Exchange

The purpose of therapeutic plasma exchange (TPE) is to remove pathological substances from the blood, such as monoclonal paraproteins and autoantibodies, as well as to replace deficient plasma components [15].

We will discuss a clinical trial followed by three cases reports regarding TPE and CCHF [13-16].

In Bestepe’s clinical trial, 119 patients with CCHF received supportive treatment (ST) or TPE. They were divided into mild, moderate, and severe CCHF groups according to the severity score index (SSI). The median SSIs were 7 in the TPE group and 5 in the ST group. Results showed that the mortality rate was lower in the TPE group than in the ST group, but the duration of hospitalization and the time to platelet recovery were longer in the TPE group. In patients with intermediate severity, the TPE was not helpful; however, it might be useful in a severe disease presentation [13].

In Ture and Kalin-Unuvar’s case report, a 61-year-old woman with CCHF and complications with thrombocytopenia (2,700 uL) received three sessions of plasma exchange through a central venous catheter, achieving adequate control of the fever. Following the sixth day of hospitalization, she developed pulmonary embolism. Treatment with enoxaparin was initiated, and on day 14, she was discharged home with rivaroxaban [14].

Meço et al. described in their case report a type of plasma exchange called double filtration plasmapheresis (DFFP) due to its superior effectiveness over TPE. A 44-year-old man with a late CCHF was treated with supportive therapy (fresh frozen plasma [FFP] and plasmapheresis) in conjunction with ribavirin therapy. Despite these therapeutic measures, the patient’s clinical signs and laboratory findings deteriorated; therefore, DFPP was performed via femoral double-lumen catheter with the aim of decreasing the viral load. After 16 days of ICU treatment, the patients’ clinical signs regressed, and laboratory parameters turned to normal values [15].

Plasmapheresis was used in Kurnaz et al. case report because a 71-year-old woman with CCHF did not improve despite ribavirin treatment. She later developed DIC, and TPE was initiated as rescue therapy. Following three sessions, her laboratory parameters improved along with ribavirin therapy, and she was discharged without complications on day 21 [16].

The clinical trial showed decreased mortality with TPE therapy, but hospitalization days and platelet recovery were longer compared to ST [13]. Additionally, in the case report where ribavirin and TPE were used, a positive outcome was seen [15]. Further studies should explore the combined treatment of oral ribavirin and plasmapheresis, especially in patients with severe disease [16]. In the other case reports, patients who received plasma exchange survived without any sequelae to their health. One case report received ribavirin along with TPE providing a good outcome as well [13,14]. This combination treatment can be an excellent tool to decrease the viral load and therefore improve recovery possibly. We can conclude that TPE can be defined as a good adjunct to conventional antiviral therapy in severe CCHF cases [13-16].

Corticosteroids

Corticosteroids decrease the production of leukotrienes and prostaglandins, halting the inflammatory cascade by decreasing leukocyte migration, capillary permeability, phagocytosis, platelet-activating factor, and interleukins [17].

Dokuzoguz et al.’s study recommends stratifying patients based on an SSI for case management. A total of 281 confirmed CCHF cases were included, of which 23 died. Ribavirin was effective in reducing mortality among moderately ill patients. In contrast, steroids were found to be beneficial among patients with more severe diseases, causing decreased production of proinflammatory cytokines [17].

In Jabbari’s case series, there were six patients aged 13 to 29 years who were admitted to the hospital with symptoms of sudden onset of fever, skin eruptions and petechiae, purpura, epistaxis, and myalgia [18]. Two of them were students, two were butchers, one was a driver, and another a housekeeper. Bleeding sites were different in all patients, but the two common laboratory findings were leukopenia and thrombocytopenia. Liver enzymes were slightly elevated. All patients were successfully cured after a treatment combination of supportive therapy, ribavirin, and corticosteroids [18].

With both studies, we can rely upon that the adjuvant use of glucocorticoids is an essential add-on for decreasing the acute inflammatory response caused by the course of the disease. Corticosteroids decrease mortality in severely ill patients in the observational study, and in the case series all the patients survived when corticosteroids were added to the currently effective known treatment, ribavirin, in the early stage [17,18].

Intravenous Immunoglobulin

CCHF is characterized by a macrophage-activating syndrome, which starts during the period when viremia decreases. A cytokine storm initiates and triggers the development of hemophagocytic lymphohistiocytosis (HLH), characterized by an overactivation of macrophages causing DIC, liver dysfunction, and endothelial damage [17,19]. Supportive therapies such as packed red blood cells, FFP, and platelet transfusions are important for CCHF, as well as close monitoring of vital signs. In the hemorrhagic phase, the primary causes are DIC, thrombocytopenia, and direct endothelial damage caused by the virus, making the patient susceptible to death [19]. The use of antiviral drugs in this phase is non-beneficial because it would not suppress the cytokine storm; therefore, treating DIC and HLH in this particular phase will be effective for decreasing mortality [19].

Erduran et al. treated 12 patients with CCHF associated with HLH. All of them underwent bone marrow aspiration that showed hemophagocytosis. High-dose methylprednisolone (HDMP) therapy was initiated for suppressing macrophage activation, FFP for treating DIC, and IVIG for the severe thrombocytopenia caused by DIC. After 10 days of treatment with HDMP, thrombocytes and leukocytes count reached a level higher than 150,000/mL and 4500/mL, respectively, and FFP treatment was stopped when previously elevated D-dimer decreased to <1 mcg/d. Negative PCR for CCHF was found in one case due to late admission and in the convalescence phase of CCHF, which raises suspicion that PCR for CCHF may be negative in this particular phase. The authors recommend that FFP and HDMP should be initiated as soon as the diagnosis is made. IVIG should be given in case of severe thrombocytopenia resistant to HDMP treatment and if petechiae and ecchymoses recur. The combination treatment of methylprednisolone, FFP, and IVIG seems to be effective for CCHF associated with HLH [19].

In Salehi et al.’s clinical trial, 40 patients diagnosed with CCHF by specific IgM and IgG antibodies by ELISA test were treated with ribavirin only and ribavirin plus IVIG. Almost all patients were in the city (infections are most common in rural areas) and were infected by close contact with animal products and infected secretions. Twelve patients randomly selected received ribavirin and IVIG (case group) and 28 received only ribavirin (control group). IVIG adjuvant therapy improved the severity of clinical signs and symptoms and reduced the duration of disease but without a difference in mortality rates in both groups (p = 0.171). The authors recommend the need for future studies for gathering more data [20].

IVIG in combination with FFP and methylprednisolone seemed to be effective in CCHF associated with HLH [19]. Similarly, the other IVIG study demonstrated improvement in symptoms and reduced disease duration when given in combination with ribavirin; however, it did not reduce mortality [20]. Therefore, both studies showed that adjuvant therapy with IVIG has an important role in decreasing the severity of clinical symptoms.

Hyperimmunoglobulin

The clinical trials of Kubar et al. showed promising results with hyperimmunoglobulin with a standard dose of 400 KU (Kubar units). The survival rate was 86% in the treatment group. Despite the small sample size, the author suggests that hyperimmunoglobulin may be helpful, especially in high-risk patients, defined by having a viral load greater than or equal to 108 copies/mL or more [21].

Supportive Treatment

Supportive therapy is an essential part of case management. Bleeding preventive measures should be considered and taken, such as the use of histamine receptor blockers for peptic ulcer patients, avoidance of intramuscular injections, and use of aspirin or other drugs that act on the coagulation system. Non-steroidal anti-inflammatory drugs should be avoided to decrease the systemic bleeding tendency. Fluid and electrolyte balance should also be monitored. Supportive therapy also includes the administration of platelets, FFP, and sometimes erythrocyte preparations. The replacement therapy with these blood products should be performed by checking complete blood count, which should be done daily [22].

FFP is indicated to replace clotting factors in patients with demonstrated deficiencies, such as a prothrombin time or partial thromboplastin time greater than 1.5 times normal or an international normalized ratio (INR) greater than 1.6. FFP is most commonly used in the setting of acquired coagulopathy, such as in patients with liver disease, DIC, or excess warfarin effect [22].

Platelet transfusion works in the prevention or resolution of bleeding caused by thrombocytopenia or platelet dysfunction. According to the author, as a general rule, platelet counts should be obtained 18-24 hours post-infusion. Because DIC occurs in the course of CCHF, some degree of platelet destruction is expected, and a rapid increase in platelet level after transfusion may not be observed [22].

## Conclusions

CCHF is a rapidly developing infection that has an unfavorable outcome if not diagnosed and treated on time. This prompts researchers to investigate possible solutions to avoid the high mortality rates accompanying the condition. Treatment with corticosteroids did show significant improvement to support its use in severe stages. Similarly, plasma exchange showed efficacy by decreasing mortality and possibly by reducing the viral load. IVIG continues to be used in other viral hemorrhagic infections, such as Ebola, with good results, and with CCHF it is no different; it showed decreased severity of symptoms and mortality. The hyperimmunoglobulin study, despite having a small sample size, also demonstrated to be helpful, especially in high-risk patients.

Antiviral plus adjuvant therapy showed the most promising results in various disease stages. Outbreaks in areas where the disease is not common, no therapeutic measure showed a favorable outcome in mortality rate due to the lack of suspicion for the disease. In general, more research should be conducted on the drugs discussed in this review to establish a definitive treatment and guidelines. This prompts the urgency of further investigation in this field.
